# Giving behavior and social decision-making in the age of conscious capitalism: A case for neuroscience

**DOI:** 10.3389/fpsyg.2023.1073632

**Published:** 2023-03-28

**Authors:** Sara Palermo

**Affiliations:** ^1^Department of Psychology, University of Turin, Turin, Italy; ^2^Neuroradiology Unit, Department of Diagnostic and Technology, Fondazione IRCCS Istituto Neurologico Carlo Besta, Milan, Italy; ^3^Istituto Nazionale di Filantropia – Filantropolis, Numana, AN, Italy

**Keywords:** conscious capitalism, leadership, stakeholders, giving behavior, altruism, social cognition, social brain, prefrontal cortex

## 1. Introduction

Giving behavior is governed by empathic, emotion-regulating and social cognition processes that support altruistic behavior, which is one of the factors that determine social interaction. According to Mauss (1925), societies have advanced to the point where they, their subgroups and finally their individuals are able to make their relationships stable thanks to giving, receiving and finally reciprocating. In this framework, the gift represents a free and unrestricted exchange through which the society emerge and social order is maintained (Mauss, 1925; Adorno, 1994). “*Cuddling for survival*” is indeed based on the idea that altruistic individuals *profit back* by investing in their social environments (Nowak, 2012).

Social welfare today is heavily influenced by altruistic and philanthropic behaviors, which are being increasingly studied in neuroscience and neuroeconomics. One reason is that the brain has variable ways of calculating, interacting, and implementing variables in economic models of altruistic choice (Hutcherson et al., 2015). Indeed, altruistic and empathic decision-making emerged from the stochastic accumulation of relative value signals that are linearly weighted based on information about self and other payoffs in *a multi-attribute attentional drift-diffusion model* (Milosavljevic et al., 2010; Morishima et al., 2012; Geoffrey Fisher, 2021; Yang and Krajbich, 2023).

Although neuroscientific knowledge comes overwhelmingly into play in the debate about the motivational drives inherent in giving behavior, the development of observational and intervention models is headed by the social and economic sciences. This article aims to encourage the use of neuroscientific methods to philanthropy and the culture of giving. The case of leadership in the evolving framework of conscious capitalism is taken as the starting point for discussion.

## 2. An economic model of the social brain and social decision-making

**Figure 1:**
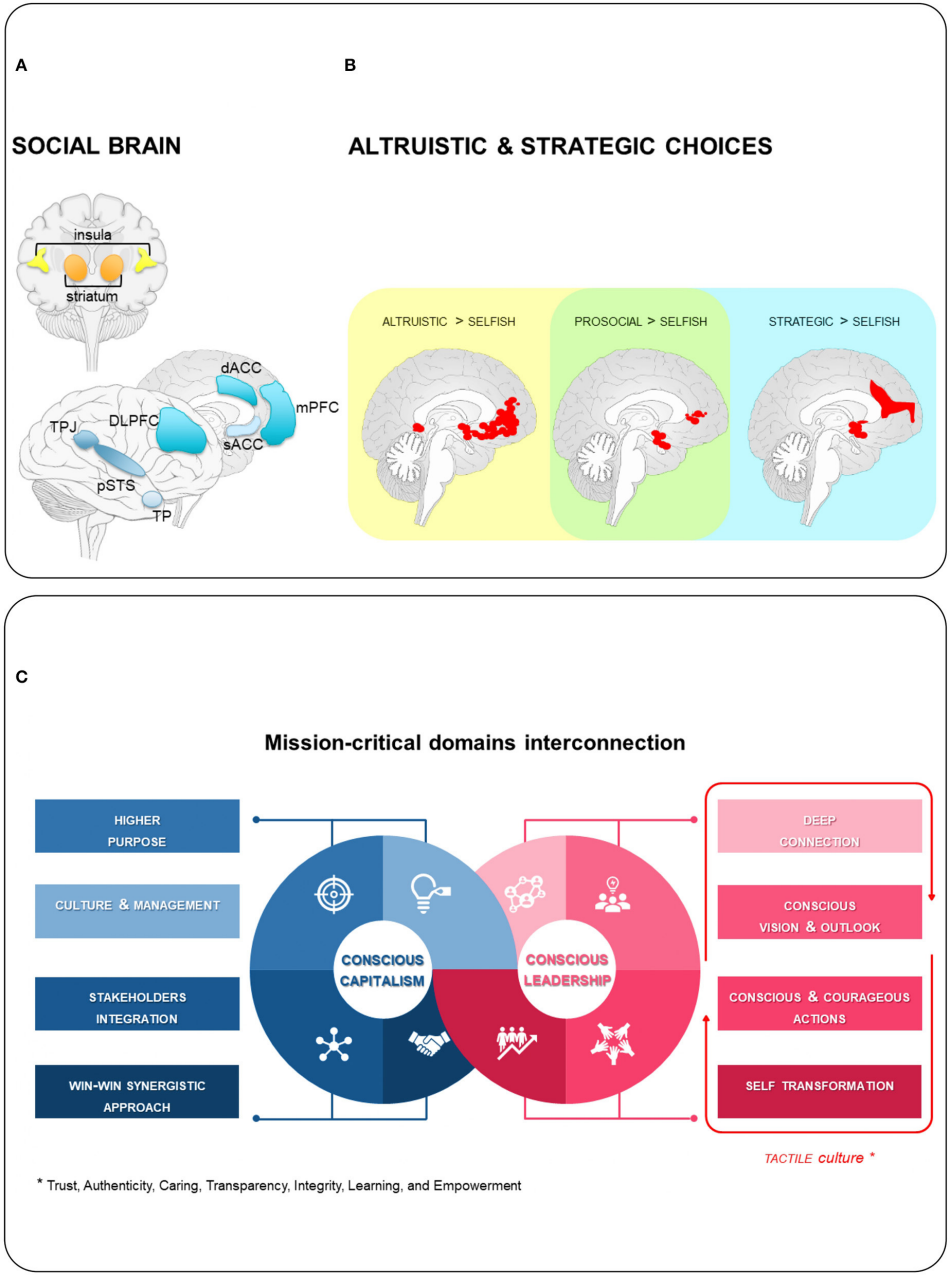
**(A)** Social brain is primarily involved in social cognition (adapted from Windmann and Hein, 2018). Main core hubs shown. TPJ, temporal parietal junction; dACC, dorsal anterior cingulate cortex; MPFC, medial prefrontal cortex; sACC, sugbenual anterior cingulate cortex; DLPFC, dorsolateral prefrontal cortex; TP, temporal pole; pSTS, posterior part of the superior temporal sulcus. **(B)** Consistent neural underpinnings of altruistic and strategic choices concerning giving behavior (adapted from Cutler and Campbell-Meiklejohn, 2019). **(C)** Conscious capitalism and leaders interconnections become the foundation of a new organization's operating philosophy. Philanthropy becomes a policy of donating part of the profit to non-profit organizations with emphasis on intrinsic motivation and contribution to social needs through the choice and the quality of goods and services. On the leadership side, there is a mutual reinforcement between vertical learning principles thanks to the TACTILE culture.

The abilities that enable people to construct mental representations of their relationships with others to adapt behavior to the context are referred to as *social cognition*, the complexity of which has resulted in such an evolution of the prefrontal brain areas that the existence of a *social brain* has been acknowledged (Windmann and Hein, 2018; Morese and Palermo, 2022) (Figure 1A).

Social decisions are a subset of economic decisions in that they need to consider both the interests of others and their own interests. Because options are compared based on subjective preferences, there are no a priori correct choices. One simply chooses the highest value option. It is usually necessary to weigh the immediate motives against the long-term consequences of one's decisions in the real social environment (Báez-Mendoza et al., 2021). In this economic perspective, good-based decision-making involves acquiring sensory information and integrating it to *external* (environmental) and *internal* (psychological) *determinants* (Báez-Mendoza et al., 2021; Terenzi et al., 2021). Frontoparietal mirror neurons and cortical midline structures play a role in the neural mechanisms underlying the influence of internal factors on social decisions (Terenzi et al., 2021). These networks bridge the gap between the self and social factors by processing and integrating information about the physical and mental self and others (Uddin et al., 2007).

As long as humans are capable of generating utility directly from alleviating the suffering of others (Andreoni, 1990), mesolimbic reward systems are activated for both monetary rewards and donations (Weiss et al., 1971; Moll et al., 2006). The ventromedial prefrontal cortex encode the value of basic rewards at the time of choice, serving as a valuation system during social decision-making (Hare et al., 2010; Hutcherson et al., 2015). Also, the medial and subgenual orbitofrontal areas, as well as the lateral orbitofrontal areas, play a key role in social attachment and adversion, specifically influencing decisions regarding *donations to* or *opposition to* social causes. Subtle differences in the architecture of specific brain circuits allow to distinguish between altruistic actions dictated by *empathy* and those inspired by a more “selfish” feeling of *reciprocity*: while information flows primarily from the anterior cingulate cortex to the anterior insula when empathy is the motivation for altruistic action, it goes in the opposite direction when reciprocity is the motivation (Hein et al., 2016).

## 3. A case study for neuroscience: The transformation of leadership in the context of conscious capitalism

*Conscious capitalism* can be listed to be a major trend in the foreseeable future. It refers to individuals who set higher goals and adopt more effective operational practices aimed at stakeholders who have a higher level of social awareness (Aburdene, 2005; Kofman, 2006; Sisodia, 2009; Sisodia et al., 2011).

The premise of conscious capitalism is that companies should pursue profits while doing business morally and considering social, cultural and environmental wellbeing, while empowering people to make a difference (Sandelands, 2009; Frémeaux and Michelson, 2017). This allows maximizing profits in the long term. Four elements are fundamental to achieve the goal: moving beyond mere profit maximization and involving the entire company in the process; participation of all stakeholders in value creation through interconnectedness; recognition of the interdependence of all business systems and the need for win–win synergistic approach; and a tangible culture of the common good (Kofman, 2006; Sisodia, 2009; Whittington, 2017).

It takes more than economic resources to exercise leadership. Identifying leadership opportunities and establishing processes for grounding decisions are all crucial to making a difference. What is important and what goals to pursue depends on the motivational construct of *value* (Brosch et al., 2011). Economically, differences in value hierarchies refer to two orthogonal dimensions: *strategic* (selfish and extrinsic rewards-based) and *altruistic* (intrinsic rewards-based) *choices* (Figure 1B). The ability to manage their mutual influence is essential for effective conscious leadership (Cutler and Campbell-Meiklejohn, 2019).

Leadership has been proven to be more effective across a variety of mission-critical domains thanks to the change of mindset enabled by *vertical learning* (Petrie, 2011; Brown, 2012a,b) (Figure 1C). Based on the above, conscious leadership consists of:

- *Deep connection*: decisions are driven by personal values/motives to which one consistently adheres.
- *Conscious vision and outlook*: intuition is supported by sophisticated tools and pipelines.
- *Conscious and courageous action*: trust in one's own and others' skills allows one to adapt flexibly to circumstances.
- *Self-transformation*: personal/skills growth is never considered complete.

## 4. A proposed neuroscientific perspective

Given the context of transformation inspired by conscious capitalism, one wonders whether the perspective of neuroscience can be expressed with benefit in such a far-from-usual context.

As a form of social capital, leadership involves the sharedness, distributedness, and connectivity of the members of the organization (Balconi, 2020). Neuroscience can help develop conscious leadership that is more self-aware and able to “tune in.” To identify the most functional modalities to regulate the relationship between leaders and employees, behavioral mechanisms of *synthonization* have been studied. Using connectivity analysis, a direct measurement of “brain tuning” can be made (Balconi, 2020): it is possible to examine both the level of neural tuning within individuals (single-brain connectivity) as well as how this connectivity increases and consolidates between the two brains (interaction analysis). Neurophysiological and neuropsychological responses can be synchronized in a variety of contexts and used to assess the coupling between two or more systems interacting (Balconi and Vanutelli, 2017).

Inter-brain connectivity occurs when individuals coordinate their actions according to shared rules when they perform complex behaviors. Brain-to-brain coupling is an unconscious process that adjusts understanding and communication between individuals during interactions (Hasson et al., 2012). This way, inter-brain connectivity promotes cooperative behaviors, empathic actions, and prosocial behaviors (Mogan et al., 2017). By increasing interactive behavioral synchrony, inter-brain connectivity acts as a neural basis for consciousness, enhancing empathy and the sense of involvement, affinity, and closeness between individuals participating in an interaction or performing a common task (Bevilacqua et al., 2019).

Neuroeconomics is interested in how leaders make trust-based decisions—particularly important in the field of philanthropy. The process of trust decision-making is largely unconscious, so neurophysiological measurements have helped to gain insights into how people make, even when they are unaware of how they do so (Balconi, 2020). Furthermore, neuroimaging research might reveal differences between leaders with a tendency to pursue bolder or riskier alternatives and those with a tendency to be more conservative or risk-averse. Ashkanasy (2003) discussed the neurological basis of the “freezing response” or the tendency to “freeze in fear.” The fear response involves links between the cortex or thalamus and limbic areas, specifically the amygdala. Risk-averse strategic decision-makers, therefore, are more likely to freeze when considering potentially bold or risky decisions, due to specific aspects of their brain activity. Another aspect of decision-making is moral judgment. The frontal cortex participates in moral judgments and evaluations of fairness. Additionally, moral emotions such as guilt, indignation, and compassion are present (Balconi, 2020). Moreover, social information about the relative popularity of a need may evoke an empathetic concern for the most disadvantaged target, consistent with the human tendency to avoid unequal distributions (Saito et al., 2019). One element that comes overwhelmingly into play is social reward. Indeed, several neural mechanisms of giving behavior can be identified in relation to reputation, which is an important aspect of social cognition. Giving behavior is based partly on the reputation of the person/organization in need. Moreover, helping others improves one's reputation and it indirectly increases the chance of receiving assistance in the future if needed (Izuma, 2012). Accordingly, individuals seek to maximize their own benefits by helping others based on altruistic-strategic choices. These are biologically non-interchangeable at the brain level. The former recruit the subgenual anterior cingulate cortex, the latter the nucleus accumbens (Cutler and Campbell-Meiklejohn, 2019). Neural correlates of altruistic-strategic choice evaluations were explored individuals freely decide whether or not to donate in the presence or absence of observers (Izuma et al., 2010). Not only the mere presence of observers increased donation rates, but it significantly altered activation in the ventral striatum before choosing whether to donate. Striatal activation was higher when a high social reward was expected (donation in public) and when monetary gain was expected with no social cost (no donation without observers) (Izuma et al., 2010). At the brain level, therefore, social and monetary rewards are represented as “decision utility,” and each choice is made using a “common neural currency” in social situations. These mechanisms cannot but be reverberated in conscious leadership as well. Clearly, giving without expecting something in return is special. According to the latest findings on altruism and philanthropy, being generous activates the brain's reward system. When exposed to a charitable rewarding stimulus, the brain responds by increasing release of the neurotransmitter dopamine. This might be a biological response to assisting others that inspires an imitative principle through mirror systems and binds societies with altruism and cooperation. Indeed, empathy involves appropriate affective response to another person's situation. Such affective response could later be translated into helping behavior. Hatfield et al. (1993) have previously discussed emotional contagion and how it is a key component of empathic processes and altruism.

## 5. Discussion

This opinion article starts from the assumption that human behaviors are motivated not only by materialistic rewards, but also by evolutionarily innate altruistic behaviors and social rewards contributing to brain ontogeny and societal development. It therefore becomes interesting to apply neuroscience methods to the case of conscious capitalism and giving behavior. Indeed, taking social actions requires the brain to translate different rewards (such as money, pleasure in contributing to the common good, and social approval) into a common scale.

Conscious capitalism transcends philanthropy, as it intends to build an entirely new structure for companies which financial integrity have to be based on “social consciousness,” which is an evolutionary mechanism that allows people to navigate multiple and complex relationships.

There is a boundary that allows mutual exchange between organizations and their components. Leadership is the regulatory function responsible for governing exchanges, located ideally along the entire boundary. All actors are inseparably involved in a co-constructed process that results in values and behaviors emerging through conscious leadership. In anthropo-psychological terms, a shift has occurred from *Homo Economicus* to *Homo Relationalis* as the species evolves together as a group rather than individually as it uses its consciousness to interact with each other's. In this shift, neuroscience and social brain come into play. There must be an immediate mechanism that causes the humans to exhibit a particular social economic behavior (Kedia et al., 2017). Neuroscience has given a new method for measuring the core psychological processes that underpin altruistic conduct and economic decision-making without relying on behavior or introspection, and this study has yielded new insights. There is a significant role for social decision-making in giving behaviors, which uses the neural mechanism of decision-making within a strictly social context (Cosmides and Tooby, 2013). Moreover, when looking for concepts of strong social desireability, objective traces of subjective motives can be very fruitful. This is precisely the case with conscious leadership. Is giving behavior necessarily caused by psychologically altruistic mechanisms? Strategic- and altruistic- choices predispose the individual to direct their giving behavior, reciprocally in an *action-* or *outcome-*oriented sense (Kuss et al., 2013). Giving behaviors are associated with recruitment of the dopaminergic reward system, providing support for positive feelings associated with the strategic (*action-oriented*) choices. At the same time, indirect support for altruistic (*outcome-oriented*) choice was found, showing an increase in reward-related brain activity during non-voluntary money transfers (Kuss et al., 2013). Action-oriented and result-oriented motivations are indeed supposed to complement each other. Not only does neuroscience enable an assessment of motivation, but it also promotes the understanding and unraveling of rewarding vs. punitive altruism. If functional activations and connectivity of the anterior insula and temporo-parietal junction play specific roles in empathic forms of altruism, subgenual anterior cingulate cortex and the nucleus accumbens in altruistic vs. strategic altruistic choices, the dorsolateral prefrontal cortex, among other regions, is involved in norm-oriented forms of punitive altruism (Windmann and Hein, 2018).

To thrive in this difficult interplay of moderator-mediators of psychological and psychobiological nature, conscious capitalism must have extraordinary leadership capabilities and delivery (Goleman, 2011). The field could be greatly benefited from neuroscience studies, beyond the increasingly popular neuroeconomics, and neuromarketing. One example is undoubtedly the “organizational cognitive neuroscience” (OCN) or neuromanagement. Another is the application of social neuroscience to the non-profit sector: can emotions go beyond igniting one-time donations and mobilizing people to participate to create long-term supporters for non-profits? Are donations driven by empathy or by a psychological response or utilitarian calculation? One of the most current challenges is to unravel the ambiguity and conflict of motivations in the pursuit of altruistic intentions.

To summarize: analogous to the “social brain,” is there a “charitable brain” responsible for giving behavior? Could “giving behavior” be a way to better understand empathy, mentalizing, and social decision-making and to explore the neural correlates underlying them? Can such knowledge be harnessed to extend the culture of conscious capitalism and enhance society? There is no shortage of areas for neuroscience intervention. One of the most important gaps to be filled is how to intercept unmet needs in the economic domain by a discipline born out of the *life sciences* and *Humanitas*. There are good opportunities for neuroscience of giving behavior.

## Author contributions

SP conceived the content of the article and personally drafted the manuscript, wrote the final version of the document, and created the infographics.
